# Consuming grass finished lamb improves blood plasma ω-3 fatty acid response among healthy consumers

**DOI:** 10.1007/s00394-025-03765-z

**Published:** 2025-07-21

**Authors:** Lynda S. Perkins, Anne P. Nugent, Jayne V. Woodside, Chris R. Cardwell, Nigel D. Scollan

**Affiliations:** 1 The Institute for Global Food Security, School of Biological Sciences, Queen’s University Belfast, 19 Chlorine Gardens, Belfast, BT9 5DL Northern Ireland, UK; 2 Institute of Food and Health, School of Agriculture and Food Science, University College Dublin, Dublin, D04 V1W8 Ireland; 3 Centre for Public Health, School of Medicine, Dentistry and Biomedical Sciences, Queen’s University Belfast, Belfast, Northern Ireland, UK

**Keywords:** Lamb, Omega-3, ω-3, Fatty acids, Plasma, Plasma phospholipids

## Abstract

**Background and purpose:**

Dietary intakes of omega-3 polyunsaturated fatty acids (ω-3 PUFA) are below what is recommended. Meat from grass finished ruminants contains higher levels of ω-3 PUFAs, particularly, alpha-linolenic acid (C18:3 ω-3). The impact of consuming grass finished lamb meat rich in ω-3 PUFA on blood fatty acid (FA) response in humans is not well established. This study investigated the impact of consuming grass finished lamb on total blood plasma and blood plasma phospholipids (PL) FA and on cardiovascular risk factors, including heart rate, blood pressure (BP), HDL, LDL, total cholesterol, and TAG, in humans.

**Methods:**

A single blinded, randomised controlled trial was conducted. Two portions of lamb chops and one portion of lamb mince from lambs finished on a grass or concentrate diet were consumed per week by 34 healthy participants for four consecutive weeks. Blood samples were taken at baseline and post-intervention. Approximately ~ 328 mg/100 g of total ω-3 PUFA was present in grass finished lamb portions per week.

**Results:**

Greater levels of ω-3 PUFA, namely C18:3 ω-3, eicosapentaenoic acid (C20:5 ω-3) and docosapentaenoic acid (C22:5 ω-3), were detected in total blood plasma from participants who consumed grass finished lamb (*P* < 0.05), while consuming concentrate finished lamb higher levels of linoleic acid (C18:2 ω-6) in plasma PL (*P* < 0.01). There were no differences of consuming grass or concentrate finished lamb on cardiovascular risk factors.

**Conclusion:**

This study demonstrates that ω-3 PUFA from lamb meat finished on grass is reflected in blood ω-3 PUFA response. Grass finished lamb is a useful matrix for increasing intake of ω-3 PUFA into the human body alongside little required change to customary dietary habits.

ClinicalTrials.gov ID: NCT06607354. Retrospectively registered 24/09/2024.

## Introduction

Ruminant meat is often criticised for containing high levels of saturated fatty acids (SFA), relative to white meat, despite being rich in protein, iron, vitamin B12 and omega-3 (ω-3) PUFA [1]. The nutritional composition of meat is greatly influenced by diet of the ruminant and the benefits of grass finishing ruminants to improve levels of ω-3 PUFA in muscle, namely C18:3 ω-3, are recognised [2–5]. More recently, grass-based feeding has been reported as a more environmentally friendly alternative to concentrate feeding [6].

Omega-3 PUFAs are beneficial to human health and well-being, with optimal intake associated with improved cardiovascular health, neurodevelopment, and reduced blood pressure (BP) [7–10]. Research has indicated that the bioavailability and effect of consuming dietary sources of ω-3 PUFA is more efficient than supplementary sources such as fish oil tablets [11]. The benefits of consuming dietary ω-3 PUFA are reflected in nutritional claims. For food products to be considered ‘a source of’ *n- 3* PUFA it must contain per 100 g and 100 kcal, 300 mg of C18:3 ω-3 or 40 mg of C20:5 and C22:6 ω-3 [12]. Similarly, for food product to be labelled ‘high in’ ω-3 PUFA it must contain per 100 g and 100 kcal, 600 mg of C18:3 ω-3 or 80 mg of C20:5 and C22:6 ω-3 [12]. UK dietary guidelines recommend that two portions of fish should be consumed per week, one of which is oily [13]. Oily fish is known to be the richest source of long chain (LC) ω-3 PUFA [14]. Dietary intake of fish in the UK is low, with 56 g/week being consumed by 19–64-year-olds [15]. Red (and processed) meat, however, is consumed at higher levels with a mean intake of 44 and 69 g/day reported for women and men (aged between 19 and 64), respectively [15]. Changing consumer behaviour is challenging, therefore, improving the nutritional value, in particular ω-3 PUFA, in red meat is an appropriate avenue to explore.

There are limited dietary intervention studies connecting consuming ω-3 PUFA enriched meat products and effects on human health. Consuming ω-3 PUFA enriched chicken and eggs resulted in greater levels of C20:5 ω-3, C22:6 ω-3 in red blood cells (RBC), in 146 consumers [16]. Similarly, one study reported a positive response in blood plasma and platelets when ω-3 PUFA enriched beef and lamb was consumed [17]. The literature suggests that grass finished lamb has a higher level of total fat and can store greater levels of ω-3 PUFA, compared to beef [18, 19]. Despite this, the effect of lamb consumption on blood FA, particularly ω-3 PUFA, bioavailability and uptake within blood plasma and blood plasma PL in humans is currently unknown.

The aim of this study was to investigate the impact of consuming grass finished, compared to concentrate finished, lamb meat on blood response and cardiovascular risk factors in humans. The primary objective was to assess the effect of consuming three portions of grass or concentrate finished lamb meat per week, for four consecutive weeks, on total blood plasma and plasma PL FA blood response of healthy individuals. The secondary objective was to assess the effect of consumption of grass finished lamb meat on cardiovascular risk factors including BP, body mass index (BMI), total cholesterol (TC), HDL, LDL and TAG among healthy volunteers.

## Experimental methods

### Ethical approval

Lambs used in this study were part of a larger study conducted at The Agri Food and Bioscience Institute (AFBI), Hillsborough, Northern Ireland. This study was approved by their research ethical review committee and all animal procedures complied with the Animals in Scientific Procedures Act (1986). The randomised controlled trial (RCT) involving human volunteers was approved by The School of Medicine, Dentistry and Biomedical Sciences Research Ethics Committee at Queen’s University Belfast (ethics number: 19.08) and follows the Declaration of Helsinki [20]. ClinicalTrials.gov ID: NCT06607354. Retrospectively registered 24/09/2024.

### Lamb study

A mixture of Easycare, Charollais, Charmoise, Dorset and Texel lambs (*n* = 53) were produced on two farms in Northern Ireland and split into two treatments, grass (*n* = 29) or concentrate (*n* = 24; with ad libitum barley straw). Lambs on the grass treatment were zero grazed on perennial ryegrass pastures, while lamb on the concentrate treatments received commercial pellets and barley straw only for the duration of the study period. Treatments balanced for sire, weight, and gender and the experimental period lasted for 12 and 8 weeks for farm 1 and farm 2, respectively. Lambs were transported from the farms to a commercial abattoir (Dunbia, Dungannon, Northern Ireland) when an average live weight of 40 kg was reached. The lamb carcases were deboned and processed into loins, legs, and shoulders. Loins from all lambs were split into individual chops. Mince portions were produced by combining muscle from deboned, trimmed legs and shoulders. The combined muscle was minced separately into the grass and concentrate treatments. After, 250 g portions were weighed out. Lamb meat used in the study was subject to appropriate food safety tests by an independent company (ALS Life Sciences, Ireland), which included total Viable Count, *E. coli*, *E. coli* 0157 and Salmonella. These tests ensured the meat was suitable and safe for human consumption.

### Meat fatty acid analysis

Samples were analysed raw using a direct bimethylation procedure [21]. Once freeze dried (~ 72 h), 0.2 g of sample was weighed out and 4 ml of 0.5 M sodium methoxide in anhydrous methanol and 1 mg/ml of C21:0 (Internal standard) dissolved in 1 ml heptane was added to the glass test tube. Samples were heated for 15 min at 50 °C. After which, 4 ml of acetyl chloride in anhydrous methanol (1:10 v/v) was added. Samples were then heated at 60 °C for 60 min, Samples were removed from the water bath and 2 ml of heptane and 2 ml of deionised water were added. The tubes were vortexed and centrifuged for 5 min at 1500 × g at 10 °C. The organic layer (upper layer) was then pipetted into a second tube. To the original tube, 2 ml of heptane was again added and vortexed, centrifuged, and organic layer pipetted out. Next, 0.2 g of sodium sulphate was added to the second tube and then vortexed. The fatty acid methyl esters (FAME) were then aliquoted into a gas chromatography (GC) vial and placed in a − 20 °C freezer for analysis.

### GC flame ionising detector (FID) analysis for meat fatty acids

Lamb FA samples were analysed using an Agilent GC-FID programme. Helium was used as a carrier gas at a rate of 2 ml/min. Separation of FAMEs was achieved by using a CP Sil 88 FAME 100 m × 0.25 mm × 0.2 µm column (Agilent, Santa Clara, California, USA). Injector volume was 1 µl, with a 10:1 split. The initial temperature programme was 70 °C held for 1 min. A ramp of 5 °C/min was applied until temperature reached 100 °C and held for two minutes. A second ramp of 10 °C/min was applied until a temperature of 175 °C was reached and held for 34 min. Ramp four included a rate of 4 °C/min until 225 °C and held for 19 min (total run time 82 min). Standards (*n* = 39) were used to identified FAMEs. Two chromatograms were created using a 37 FAME mix and individual C18:1 *t11* and 22:5 ω-3 FAMEs (Supelco 37 FAME, Supelco cis-7,10,13,16,19-Docosapentenoic acid methyl ester and Supelco *trans-11*-Vaccenic methyl ester). Individual four-point calibration curves were created for the 39 FAMEs, in addition to the internal standard (C21:0), which enabled quantification into mg/100 g FAMEs. After, mg/100 g FAMEs were converted to FAs using known molecular weights as described by Møller [22]. A quality control sample was used throughout the FA analysis to confirm within and between assay precision.

### Volunteer recruitment

Potential volunteers were given an information sheet describing the study aims, participant involvement and data handling. After, potential volunteers were assessed for eligibility using a short screening questionnaire which set out specific inclusion and exclusion criteria to identify volunteer suitability. Healthy male and female non-smoking volunteers aged between 18 and 64 years were considered for participation. Participants with a BMI of < 18.5 km/m^2^ and greater than > 35 km/m^2^ were not eligible to participate. Those who took prescribed medication, including statins to reduce LDL cholesterol, and any form of dietary supplements were not eligible. Participants who consumed more than two portions of fish per month were also not eligible.

### Study design

The study consisted of a four-week, randomized dietary intervention where participants were required to eat three portions per week of lamb from one of two treatments, grass or concentrate finished meat. Lamb was substituted for habitual red meat consumption, which was determined through the screening questionnaire, and therefore participant’s habitual red meat intakes were maintained. Participants were asked to follow habitual eating for all other foods. Lamb portions included one portion of mince (raw weight 250 g) and two portions of lamb chops (raw muscle weight 235 g) per week. Raw lamb portions totalled 720 g per week. Cooking losses represented approximately 32% [23], resulting in ~ 490 g of lamb consumed, lower than recommendations by The World Cancer Research Fund [24] to consume less than 500 g of red meat per week. Approximately, ~ 328 mg/100 g of total ω-3 PUFA, made up of ~ 199 and ~ 129 mg/100 g of C18:3 ω-3 and LC ω-3 PUFA, respectively, was present in grass finished lamb portions per 100 g. The study was single blinded meaning the researchers were aware what lamb treatment participants were receiving, however participants were not.

#### Cardiovascular disease (CVD) risk factors (BMI and blood pressure (BP))

Height (cm) and BW (kg) measurements were taken at baseline and post-intervention. From this BMI was calculated. Serum TC, HDL cholesterol and TAG were measured at baseline and post-intervention directly using a “Randox Daytona Rx+” automated analyser. All samples were analysed in duplicate, and an average taken. LDL-cholesterol was calculated indirectly using the Friedewald equation [25].

### Dietary assessment

Intakes of dietary FA were assessed using a three-day food diary at week 0 and week 3 of the study. The diaries were used to assess dietary intake as well as to ensure compliance to the study conditions. Participants were required to record everything that they had eaten or drank within any three days of week 0 and week 3. There was no recommendation whether the food diary should be completed on weekdays or weekends. The information from the food diaries was inputted into Nutritics software where FA intake values were determined [26].

### Blood collection

Blood samples were taken at the Centre for Public Health, Queen’s University Belfast at baseline (week 0) and post dietary intervention (week 4). Participants were asked to fast for 12 h prior to the blood samples being taken. Three 6 ml EDTA tubes were used to collect blood for plasma separation and one 6 ml serum vacuum tube was used to collect blood for separation of serum. After blood draw, the EDTA and serum tubes were stored at 4 °C and room temperature respectively, in the dark for 40 min. The tubes were then centrifuged at 3000 RPM at 4 °C for 15 min. Plasma and serum samples were then aliquoted and frozen at −  80 °C until analysis.

### Blood plasma fatty acids

Lipids were isolated using an adaption of the Folch method [27] and FAMEs were prepared using the Morrison method [28]. Plasma (500 μl) was thawed and 6 ml of chloroform: methanol (2:1) containing 10 mg% butylated hydroxytoluene (BHT) was added. The solution was extracted for 30 min. Next, 2 ml of 0.2% calcium chloride was added. The solution was vortexed and then centrifuged at 2000 RPM for 10 min. The lower lipid layer was transferred into a clean glass tube and evaporated under nitrogen. After, 2 ml of boron trifluoride methanol was added. The solution was vortexed prior to being placed in a heat bath for 30 min at 60 °C. Once the samples cooled, 2 ml of petroleum spirit and 2 ml distilled water were added. The upper layer was transfer into a pre-weighed GC vial and evaporated under nitrogen. The remaining extract was reconstituted and dissolved in 250 µl petroleum spirit containing 10 mg % BHT/mg of extract. The samples were then placed in a − 20 °C freezer for FAME analysis the following day using GC-FID.

### Blood plasma phospholipids fatty acids

Lipids were isolated using the Folch method [27] and FAMEs were prepared using the Morrison method [28]. Plasma samples (500 μl) were thawed and 6 ml of chloroform: methanol (2:1) containing 10 mg% BHT were added. The solution was extracted for 30 min. After, 2 ml of 0.2% calcium chloride was also added. The solution was vortexed and then centrifuged at 2000 RPM for 10 min. The lower lipid layer was transferred into a clean glass tube and evaporated under a nitrogen stream. To separate the PL, 1 ml of chloroform was added, vortexed and the eluate collected into a 15 ml glass tube using Bond Elut Cartridges under vacuum (Varian NH2). The eluate was exposed to two X 1 ml chloroform washes under vacuum after which the eluate was discarded. 1 ml chloroform: methanol (60:40) was added under a vacuum. After, 1 ml of methanol was also added under the vacuum. The eluate was then evaporated to dryness using a nitrogen stream. Boron trifluoride methanol (2 ml) was added, the solution vortexed and heated at 60 °C for 30 min. Once cooled, 2 ml of petroleum spirit and 2 ml of distilled water was added. The top layer was transferred into a reweighed glass GC vial and then evaporated using a nitrogen stream. The residue was then reconstituted with petroleum spirit which contained 10 mg% BHT and 250 µl petroleum spirit/mg of extract. The samples were vortexed and transferred into a clean GC vial ready for FAME analysis.

### GC-FID analysis for blood plasma and blood plasma phospholipids

The temperature programme commenced at 100 °C, held for four minutes. A ramp of 25 °C/min was applied until the oven temperature reached 150 °C and held for eight minutes. A second ramp was then applied at a rate of 5 °C/min until 230 °C and held for 15 min. The temperature was then held at 230 °C for the remainder of the programme (45 min). Compounds separated within the GC column were passed through the FID using nitrogen as a make-up gas. Outputs were analysed using ‘Chemstation’ (MSD E.02.02.1413) software. A four-point calibration curve was created for all 39 peaks identified. This consisted of 37 from a FAME standard mix (Supelco 37 FAME mix) and C22:5 ω-3 and 18:1 *t11* individually (Supelco cis-7,10,13,16,19-Docosapentenoic acid methyl ester and Supelco *trans-11*-Vaccenic methyl ester). A quality control sample was used throughout the FA analysis to confirm within and between assay precision.

### Statistical analysis and sample size justification

Sample size was determined using power calculations based on changes in blood plasma and platelet PUFA levels from a similar study [17]. Based upon the SD of plasma PUFA of 2 units (%), with 16 in each treatment there was over 80% power to detect, as statistically significant at the 5% levels, a difference in mean PUFA of 2 units in the grass-fed and concentrate-fed lamb meat group. This difference in plasma PUFA is similar to McAfee et al. [17], however, the present study uses lamb meat only which is documented to store higher levels of ω-3 PUFA [29], compared to beef, and therefore it was expected that this would have a greater influence on blood plasma PUFA status. In addition to the 32 required participants established by statistical power calculations, two additional participants were recruited to account for participant withdrawal. Healthy volunteers (*n* = 34) were recruited and allocated to one of two treatments (*n* = 17/treatment) using a computer-generated randomization tool.

All data were tested for approximate normality prior to statistical analysis using SPSS (IBM SPSS, version 27). Lamb chops and mince composition data were both analysed using an independent samples t-test to compare the means of the meat from grass and concentrate treatments. Baseline characteristics were analysed using an independent samples t-test to assess differences between participants allocated into the grass and concentrate finished lamb treatments. Week 0 FA intakes were analysed using an independent samples t-test with week 3 data analysed using ANCOVA, with week 0 being included as a co-variate. Cardiovascular risk factors, including cholesterol, and blood plasma and blood plasma PL baseline data were analysed initially using a t-test. Post-intervention data were analysed using ANCOVA, with baseline being included as co-variate along with dietary treatment. The estimate for participant treatments is the difference between groups adjusted for baseline.

## Results

### Meat fatty acid composition

Total FA levels were higher in grass finished treatment (*P* < 0.05; Table 1). Levels of C18:3 ω-3, C20:5 ω-3, C22:5 ω-3 and C22:6 ω-3 were all higher in lamb chops from lambs were finished on grass (*P* < 0.001). Levels of C18:1 *t11* (*P* < 0.05) and C18:1 n-9 cis (*P* < 0.01) were also greater in the meat from grass finished lambs. Concentrate fed lambs had greater levels of C18:2 ω-6, C20:4 ω-6 and total ω-6 PUFA (*P* < 0.001). No difference between meat from grass or concentrate treatments were noted for C12:0, C14:0, C16:0. Total monounsaturated fatty acids (MUFA), PUFA, total ω-3, LC ω-3 PUFA, ω-6/ω-3 and PUFA/SFA were significantly higher in the grass relative to concentrate fed lamb (*P* < 0.001).

**Table 1:** Effect of grass or concentrate feeding on FA profile of uncooked lamb chops and mince (mg/100 g)

Fatty acids	Lamb chops					Mince				
	Grass (n = 29)		Concentrate (n = 24)			Grass (n = 20)		Concentrate (n = 20)		
	Mean	SD	Mean	SD	P*	Mean	SD	Mean	SD	P*
C12:0	8.4	3.66	6.4	3.91	0.067	21.6	3.77	19.3	2.589	0.030*
C14:0	106.3	37.84	87.1	37.10	0.070	222.4	31.73	234.2	37.23	0.291
C16:0	752.8	179.51	727.5	203.13	0.633	1279	159.41	1590	300.65	< 0.001*
C18:0	581.2	128.30	453.2	151.13	0.002*	1055	180.05	1095	201.21	0.516
C18:1 *t11*	133.7	73.16	90.7	65.66	0.030*	201.2	36.18	352.6	99.76	< 0.001*
C18:1 ω-9 cis	1133.2	382.73	844.9	606.89	0.040*	1710	179.68	2008	360.78	0.002*
C18:2 ω-6	61.6	17.08	159.3	42.45	< 0.001*	86.6	7.01	343.0	59.29	< 0.001*
C20:4 ω-6	21.5	4.21	31.8	9.56	< 0.001*	21.2	1.75	48.6	6.53	< 0.001*
C18:3 ω-3	56.2	15.63	21.3	10.07	< 0.001*	86.2	14.67	41.7	6.87	< 0.001*
C20:5 ω-3	16.8	3.61	8.7	2.91	< 0.001*	16.2	2.46	11.4	1.64	< 0.001*
C22:5 ω-3	19.5	3.33	13	3.29	< 0.001*	20.0	1.94	18.2	2.24	0.008*
C22:6 ω-3	6.1	1.99	4.2	2.62	0.005*	7.1	0.69	7.2	1.64	0.947
Total SFA	1509	319.81	1330	357.50	0.060	2710	377.39	3083	536.85	0.019*
Total MUFA	1290	442.68	954.3	651.36	0.030*	2000	207.52	2469	469.51	< 0.001*
Total PUFA	195.3	40.04	248.7	55.34	< 0.001*	244.3	23.09	484.9	79.72	< 0.001*
Total ω-3	99.0	20.74	47.5	16.36	< 0.001*	130.1	15.93	79.3	12.04	< 0.001*
LC ω-3	42.4	7.59	25.9	7.85	< 0.001*	43.9	4.61	37.7	5.50	< 0.001*
Total ω-6	96.3	22.14	201.2	46.94	< 0.001*	114.2	7.96	405.6	67.81	< 0.001*
ω-6/ω-3	0.97	0.24	4.24	0.08	< 0.001*	0.88	0.07	5.49	0.03	< 0.001*
PUFA/SFA	0.13	0.20	0.19	0.03	< 0.001*	0.09	0.01	0.16	0.01	< 0.001*
Total	2995	743.84	2533.2	928.12	0.050*	4955.3	595.81	6037.6	1108.28	< 0.001*

P*—Significance between treatment means using an independent t-test

Total SFA: Σ C6, C8 C10, C11, C12, C13, C14, C15, C16, C18, C20, C22, C23

Total MUFA: Σ C14:1, C15:1, C16:1c9, C17:1, C18:1ω-9t, C18:1n9c, C18:1 *t11*, C20:1 ω-9, C22:1 ω-9, C24:1 ω-9

Total PUFA: Σ C18:2t ω-6, C18:2c ω-6, C18:3 ω-6, C18:3 ω-3, C20:2, C20:4 ω-6, C20:5 ω-3, C22:2, C22:5 ω-3, C22:6 ω-3,

Total ω-3: Σ C18:3, C20:4, C20:5, C22:5, C22:6 LC ω-3: Σ C20:5, C22:5, C22:6

Total ω-6: Σ C18:2t, C18:2c, C18:3, C20:2, C22:2. C20:4

ω-6/ω-3: calculated by dividing total ω-6 by total ω-3 PUFA/SFA: calculated by dividing total PUFA by total SFA

The FA profile of lamb mince portions was significantly different between the grass and concentrate finished treatments (Table 1). Levels of SFA were higher in lamb mince, compared to lamb chops, with higher levels of C12:0 and C16:0 noted. There was, however, no effect of treatment on C18:0. Levels of C18:1 *t11* and C18:1 n-9 *cis* were higher in the lamb mince from concentrate in addition to C18:2 and C20:4 ω-6. Meat from grass finished lambs had higher ω-3 PUFAs, namely C18:3 ω-3, C20:5 ω-3, C22:5 ω-3, (*P* < 0.001), except for C22:6 ω-3. Total SFA (*P* < 0.05), MUFA, PUFA and ω-6 PUFA were greater in mince from lamb fed on concentrate (*P* < 0.001). Total ω-3 and LC ω-3, ω-3/ω-6 and PUFA/SFA ratios were higher in the mince from grass fed lamb. Total FA were higher in mince from concentrate relative to grass treatment (*P* < 0.001).

**Table 2:** Baseline characteristics for study participants (n = 34) in the participant group consuming grass and concentrate lamb

Characteristics	Grass (n = 17)		Concentrate (n = 17)		P*
	Mean	SD	Mean	SD	
Female (n = 15)	8 (23.5%)		7 (20.6%)		
Male (n = 19)	9 (26.5%)		10 (29.4%)		
Age range	22–56		21–56		
Age (years)	32.4	8.57	32.4	10.36	0.986
Height (cm)	170.1	7.39	176.7	8.78	0.025*
Weight (kg)	75.7	13.27	85.8	16.65	0.063
BMI (kg/m^2^)	26.0	3.31	28.2	4.56	0.200
SBP (mmHg)	122.2	17.66	123.8	11.17	0.753
DBP (mmHg)	79.0	8.65	85.1	9.77	0.065
TC (mmol/l)	5.2	0.74	4.8	0.64	0.101
LDL (mmol/l)	3.2	0.61	2.9	0.56	0.084
HDL (mmol/l)	1.5	0.25	1.5	0.31	0.995
TAG (mmol/l)	1.1	0.73	1.0	0.42	0.654

P*—Significance between treatment means using an independent t-test

### Baseline characteristics

All participants successfully completed the study. Of the 34 volunteers included in the study, 15 were female, with eight and seven in the participant group consuming grass and concentrate, respectively (Table 2). There were 19 male volunteers, with nine and ten in the participant group consuming grass and concentrate, respectively. The age of participants ranged from 21 to 56 years. Baseline characteristics were analysed to assess differences between the two dietary treatments (Table 2). Participants in the concentrate group were of greater height than participants in the grass treatment (*P* < 0.05). There were no differences between the participant group consuming grass and concentrate at baseline for all other characteristics, including age, weight, BMI, TC, LDL, HDL and TAG.

### Dietary assessment

There was no difference in dietary FA intakes between in the participant group consuming grass and concentrate at week 3 for C18:2 ω-6, total PUFA, total MUFA, total SFA and total FA (Table 3). However, statistically significant increases in intake relative to baseline at week 3 in the in the participant group consuming grass compared to concentrate were detected for C18:3 ω-3, C20:5 ω-3, C22:6 ω-3 and total ω-3.

**Table 3:** Intake of dietary total FA, PUFA, MUFA, SFA (g/day) at baseline (week 0) and week 3 as reported in participant food diaries

Fatty acids	Grass (n = 15)				Concentrate (n = 15)				P*	P* ANCOVA
	Baseline	SD	Post-intervention	SD	Baseline	SD	Post-intervention	SD		
Total PUFA (g/day)	8.5	4.18	7.6	2.34	8.5	4.29	9.2	4.47	0.986	0.222
Total MUFA (g/day)	21.7	7.23	21.0	5.81	19.0	9.34	23.2	11.52	0.408	0.226
Total SFA (g/day)	32.7	13.39	26.9	10.97	28.9	11.00	33.8	12.81	0.412	0.060
Total (g/day)	62.9	22.12	55.5	15.40	56.4	17.93	66.2	24.31	0.402	0.078

P*—Significance between treatment means at baseline using independent t-test

P* ANCOVA—Significance between treatment means at post-intervention using ANCOVA

Total: Σ MUFA, PUFA, SFA

### Plasma and plasma phospholipid fatty acids

There was no difference between the participant group consuming grass and concentrate at baseline for all blood FA quantified (Table 4). Levels of C18:1 n-9 *cis*, C18:3 ω-3, C20:5 ω-3 and C22:5 ω-3 FA (%) were higher in the grass compared to the concentrate treatment at endpoint (*P* < 0.05). Contrary, C18:2 ω-6 was higher in the concentrate treatment, relative to the grass treatment at post-intervention (*P* < 0.003). There were no differences in C22:6 ω-3 between grass and concentrate post-intervention. There were no differences documented for total ω-3 PUFA between treatments. Differences at post-intervention FA (%) between treatments were also detected in the blood plasma PL (Table 5). After the four-week intervention there were significant changes in C18:1 n-9 *cis*, C18:2 ω-6, C18:3 ω-3 and C22:5 ω-3. Total ω-3 PUFA was not significantly higher in the grass relative to concentrate treatment.

**Table 4:** Baseline and post-intervention human blood plasma FA composition for in the participant group consuming grass or concentrate finished lamb (%)

Fatty acids	Grass (n = 17)				Concentrate (n = 17)				P*	P* ANCOVA
	Baseline	SD	Post-intervention	SD	Baseline	SD	Post-intervention	SD		
C16:0	22.2	2.02	22.6	2.29	22.5	1.43	21.2	1.66	0.251	0.123
C18:0	9.3	1.15	8.9	1.26	9.6	0.79	9.1	0.81	0.370	0.931
C18:1 ω-9 *cis*	16.7	2.04	17.2	2.37	17.7	1.94	16.7	2.07	0.152	0.029*
C18:2 ω-6	22.2	2.94	20.5	2.41	24.1	2.55	23.7	2.23	0.052	0.003*
C18:3 ω-3	0.72	0.16	0.83	0.13	0.84	0.29	0.77	0.26	0.125	0.025*
C20:4 ω-6	6.51	1.34	6.27	0.98	5.95	0.53	5.93	0.64	0.120	0.732
C20:5 ω-3	0.74	0.20	0.85	0.21	0.66	0.21	0.68	0.20	0.267	0.034*
C22:5 ω-3	0.73	0.18	0.80	0.26	0.71	0.16	0.65	0.13	0.699	0.044*
C22:6 ω-3	1.7	0.78	1.9	0.92	2.0	0.91	1.9	0.87	0.422	0.443
LC ω-3	3.1	0.86	3.3	0.84	3.2	0.88	3.1	0.98	0.569	0.330
Total ω-3	3.9	0.93	4.1	0.98	4.2	1.00	4.1	1.03	0.306	0.287

P*—Significance between treatment means at baseline using an independent t-test

P* ANCOVA—Significance between treatment means at post-intervention using ANCOVA

LC ω-3: Σ C20:5, C22:5, C22:6

Total ω-3: Σ C18:3, C20:5, C22:5, C22:6

**Table 5:** Baseline and post-intervention blood plasma PL FA composition for in the participant group consuming grass or concentrate finished lamb (%)

Fatty acids	Grass (n = 17)				Concentrate (n = 17)				P*	P* ANCOVA
	Baseline	SD	Post-intervention	SD	Baseline	SD	Post-intervention	SD		
C16:0	29.4	1.99	29.0	1.99	28.4	1.47	27.9	1.20	0.100	0.241
C18:0	12.4	1.21	12.1	1.33	12.9	0.80	12.8	0.91	0.144	0.455
C18:1 ω-9 *cis*	12.0	2.39	12.7	1.17	11.8	1.26	11.8	0.87	0.650	0.009*
C18:2 ω-6	22.3	2.54	21.2	2.12	23.5	2.46	23.9	2.49	0.136	0.008*
C18:3 ω-3	0.26	0.06	0.34	0.07	0.30	0.08	0.27	0.11	0.139	0.014*
C20:4 ω-6	9.14	1.59	8.94	1.71	9.34	1.98	9.42	2.05	0.747	0.319
C20:5 ω-3	0.91	0.26	1.06	0.32	0.84	0.26	0.87	0.31	0.442	0.145
C22:5 ω-3	0.77	0.21	0.81	0.13	0.73	0.15	0.71	0.10	0.486	0.025*
C22:6 ω-3	2.8	0.63	2.8	0.68	2.5	0.42	2.5	0.53	0.101	0.644
LC ω-3	4.4	0.68	4.7	0.74	4.2	0.56	4.2	0.78	0.401	0.142
Total ω-3	4.6	0.71	5.1	0.75	4.4	0.74	4.4	0.84	0.146	0.148

P*—Significance between treatment means at baseline using an independent t-test

P* ANCOVA—Significance between treatment means at post-intervention using ANCOVA

LC ω-3: Σ C20:5, C22:5, C22:6

Total ω-3: Σ C18:3, C20:5, C22:5, C22:6

### Cardiovascular risk factors

Diet did not affect weight or BMI (Table 6). Similarly, there was no effect on BP at post-intervention, for both systolic blood pressure (SBP) and DBP and on TC, LDL, HDL or TAG levels (Table 6).

**Table 6:** Effect of grass or concentrate lamb treatment on human cardiovascular risk factors at baseline and post-intervention

Characteristics	Grass (n = 17)				Concentrate (n = 17)				P*	P* ANCOVA
	Baseline	SD	Post-intervention	SD	Baseline	SD	Post-intervention	SD		
Weight (kg)	75.7	13.27	75.5	12.96	85.8	16.65	86.0	16.56	0.063	0.223
BMI (km/m^2^)	26.0	3.31	26.0	3.30	28.2	5.952	28.3	6.02	0.200	0.309
SBP (mmHg)	122.2	17.66	121.8	15.45	123.8	11.17	121.1	13.54	0.753	0.521
DBP (mmHg)	79.0	8.65	81.9	10.70	85.1	9.77	80.9	9.51	0.065	0.066
TC (mmol/l)	5.2	0.74	5.4	0.71	4.8	0.64	4.9	0.73	0.101	0.221
LDL (mmol/l)	3.2	0.61	3.4	0.67	2.9	0.56	2.9	0.58	0.084	0.317
HDL (mmol/l)	1.5	0.25	1.5	0.29	1.5	0.31	1.5	0.38	0.995	0.996
TAG (mmol/l)	1.1	0.73	1.2	0.49	1.04	0.42	0.97	0.60	0.654	0.085

P*—Significance between treatment means at baseline using an independent t-test

P* ANCOVA—Significance between treatment means at post-intervention using ANCOVA

## Discussion

The study assessed the impact of consuming three portions of grass or concentrate finished lamb meat per week for four consecutive weeks on total blood plasma and blood plasma PL FA concentrations from healthy participants. The FA composition of the lamb meat was different between grass and concentrate treatments (Table 1). Grass is naturally richer in C18:3 ω-3, relative to concentrate feeding. This was reflected in higher n-3 PUFA status in participants who consumed the grass-fed lamb within dietary limits. This shows the potential for meat from grass-based feeding systems to positively contribute to circulating ω-3 PUFA concentrations in healthy adults and without adverse impacts on other health outcomes.

Pasture feeding is often associated with lower total levels of lipid reflecting lower levels of dry matter (DM) and energy intake relative to concentrate-based systems [30–32]. The concentration of C18:3 ω-3 in the lamb chops and mince was higher in grass relative to concentrate finished lamb and is supported by Boughalmi and Araba [33], Gruffat et al. [34] and Wang et al. [35]. In the present study, greater levels of C18:3 ω-3 in grass fed meat were associated with higher levels of C18:3 ω-3 in grass relative to higher C18:2 ω-6 in cereal-concentrate fed lamb. Grass finished lamb chops and mince had higher amounts of LC ω-3 PUFA including C20:5 ω-3, C22:5 ω-3 and C22:6 ω-3, reflecting the synthesis of these longer chain FA through elongation and desaturation of C18:3 ω-3 [36]. Greater levels of LC ω-3 PUFA in meat from the grass finished treatment are consistent with published literature [34, 37, 38]. In the present study, ~ 328 mg/100 g of total ω-3 PUFA, made up of ~ 199 and ~ 129 mg/100 g of C18:3 ω-3 and LC ω-3 PUFA, respectively, was present in grass finished lamb portions per 100 g. This is greater than McAfee et al. [17] whereby beef and lamb combined delivered ~ 247 mg/100 g of total ω-3 PUFA, made up of ~ 156 and ~ 91 mg/100 g of C18:3 ω-3 and LC ω-3 PUFA, respectively, per 100 g. The greater ω-3 PUFA in the lamb in this study reflects higher total fat in lamb relative to beef.

Positive changes in circulating FA proportions including C18:3 ω-3, C20:5 ω-3 and C22:5 ω-3 were observed, with no impact on cardiovascular risk factors. This supports the hypothesis that substituting red meat for grass finished lamb can contribute to enhancing ω-3 PUFA levels in human plasma (short term indicator) and plasma PL (longer term indicator) [39]. Despite limited literature, the greater levels of C18:3 ω-3 in blood plasma observed here is consistent with a similar study which compared grass finished and concentrate finished beef and lamb in a dietary intervention [17]. Incorporation of C18:3 ω-3 into the PL fraction was also noted here, with greater levels reported in the participant group consuming grass finished lamb and lower levels reported in the participant group consuming concentrate finished lamb. This further confirms the bioavailability of ω-3 PUFA from grass finished lamb, while demonstrating the benefits of regular consumption on total blood plasma and blood plasma PL response. Levels of C20:5 ω-3 and C22:5 ω-3 in blood plasma were greater in participants from the grass treatment. Greater levels of C20:5 ω-3 and C22:5 ω-3 were reported compared to a similar study [17]. This reflects the higher composition levels of C20:5 ω-3 and C22:5 ω-3 in the lamb compared to the diets provided by McAfee et al. [17]. Differences were detected in C22:5 ω-3 in the participant group consuming grass for blood plasma PL, C20:5 ω-3 was also higher, albeit not significantly. Within blood plasma, dietary C22:5 ω-3 is primarily and rapidly deposited into the PL fraction, [40, 41]. Consuming lean beef has been shown to significantly increase levels of C20:5 ω-3 and C22:5 ω-3 in plasma PL [42]. This may explain why a stronger result was detected in plasma PL, compared to total plasma. There was no effect of diet on concentration of C22:6 ω-3 in blood plasma. The rate of conversion of C22:6 ω-3 from C18:3 ω-3, C20:5 ω-3 and C22:5 ω-3, through elongation and desaturation processes is small and therefore unlikely to make a biological impact [29]. This was not mirrored in the literature, where greater levels of C22:6 ω-3 were reported when humans consumed grass finished beef and lamb over the same time period [17], despite the meat containing comparatively lower levels of C22:6 ω-3. This may be because of bioavailability differences between beef and lamb. Alternatively, fasting time from last meal directly influences FA composition in blood plasma [39]. Grass finished lamb had no effect on C22:6 ω-3 concentration in blood plasma PL. This suggests that the study duration was not long enough for incorporation of C22:6 ω-3 into blood plasma PL. There were lower amounts of C18:2 ω-6 in blood plasma and plasma PL in the participant group consuming grass finished lamb, relative to the concentrate finished lamb. This was anticipated as grass has considerably lower levels of C18:2 ω-6, compared to concentrate where it is a dominant FA [43]. Dietary treatment had no effect on LC and total ω-3 PUFA in blood plasma or plasma PL. A previous RCT of the same duration, where volunteers consumed grass or concentrate finished beef and lamb, reported differences in LC ω-3 PUFA between treatment in blood plasma [17].

There was no effect of diet on cardiovascular risk factors post intervention between the participant group consuming grass or concentrate. Specifically, there were no differences in weight (kg) or BMI post-intervention when adjusted baseline between treatments. This study suggests that consuming three portions of lamb per week for four consecutive weeks, as a part of a balanced diet had no negative effect on cardiovascular risk factor response including weight, BMI, or risk of obesity. Nevertheless, the study was not statistically powered to test the true effect of secondary parameters such as BMI, BP, or cholesterol. The study had no effect on TC in the grass or the concentrate treatment. Individual components which make up TC e.g., TAG, HDL, and LDL, however, are influenced by different time scales and varying nutritional make up of dietary products. There were no differences in LDL levels post intervention between the participant group consuming grass or concentrate. LDL are labelled as ‘bad fats’ which contribute to atherosclerosis development, increasing risk of blood clots and myocardial infarction [44]. There was no effect of dietary treatment on HDL, which is recognised as a beneficial cholesterol and greater levels are associated with atheroprotective properties [45]. There was no effect of dietary treatment on serum TAG levels. No changes to SBP and DBP both between and within treatments. Omega-3 intake of > 2 g/ is noted to lower DBP [8].

This study has demonstrated that participants consuming grass-finished, compared to concentrate-finished, lamb results in a greater dietary intake of ω-3 PUFA. Lamb production systems are associated with a lower environmental footprint relative to beef production systems [46, 47]. In addition, there is evidence to suggest that grass feeding lamb is a more environmentally friendly alternative to concentrate-feeding with lower carbon emissions [6]. Therefore, it is suggested that consuming grass-finished lamb is a more sustainable option, compared to other production systems. Grass fed lamb consumed as part of a balanced diet can play a small but significant contribution to the recommended intake of omega-3 PUFAs. Dietary consumer behavioural change is challenging, therefore, the consumption of lamb meat during this study was as a replacement for habitual meat intake and therefore meat consumption levels would remain the same as usual dietary habits and be no higher than the WCRF recommendation to consume no more than 500 g/week of red meat [24]. Lamb intake and study compliance was measured independently by the research team through meat recording diaries, whereby participants detailed how much lamb was consumed (including subcutaneous fat), what portions it was eaten in and how it was cooked. Weekly meetings between the research team and the participants as well as the completion of food diaries indicated that participants adhered to the intervention.

Strengths of this study include uniquely establishing the effect of consuming grass or concentrate finished lamb on a comprehensive range of FA. The study is a new contribution to a limited pool of published studies [17, 42]. Lamb portions were cooked in a home setting with volunteers encouraged to follow habitual cooking methods. Whilst this shows an accurate reflection of consumer culinary preferences, cooking preparation, in addition to cooking method, greatly affects the composition of the FA consumed [48, 49]. While this can be construed as a limitation due to the amount of FAs consumed being difficult to predict, it does, however, reflect customary consumer habits. Furthermore, a limitation of the study was its duration. Due to the study period being four weeks, the omega-3 index could not be analysed. The study length was four weeks to ensure a balance between participant compliance and ensuring sufficient time has passed so the ω-3 PUFA intake could be reflected in blood. RBC are a longer-term indicator of ω-3 PUFA presence (6 + weeks) [39], however blood plasma PL acts as a comparable indicator. Despite this, plasma PL are dense in phosphatidylcholines and can be considered a bias representation of long-term FA profiles [39]. Dietary intervention studies which are of greater length would be beneficial to enable ω-3 PUFA index assessment. A greater number of study participants would also allow for stronger statistical power to detect changes in cardiovascular risk factors including TC, LDL, HDL and TAG.

## Conclusion

This study has demonstrated that meat from lambs produced on grass results in greater levels of ω-3 PUFA levels in muscle, compared to concentrate. When human participants consumed three portions of grass or concentrate finished lamb meat for four consecutive weeks levels of C18:3 ω-3, C20:5 ω-3 and C22:5 ω-3 in blood plasma and C18:2 ω-6, C18:3 ω-3 and C22:5 ω-3 in blood plasma PL were higher in the participant group consuming grass finished lamb. There was no difference between participant groups consuming grass or concentrate-finished lamb on cardiovascular risk factors post-intervention when adjusted for baseline. This study demonstrates that consumption of ω-3 PUFA from lamb finished on grass, compared to concentrate, is greater in blood plasma and blood plasma PL in humans. Consuming grass finished lamb is a lower carbon footprint alternative to concentrate-finished lamb which can provide valuable ω-3 PUFA to promote health and well-being with no change in levels of meat consumption and little change to customary dietary habits.
